# Current Status of Acceptance of Disability and the Correlation With the Life Quality in Parkinson's Disease in Southwest China

**DOI:** 10.3389/fmed.2021.767215

**Published:** 2022-01-18

**Authors:** Yan Liang, Dezhi Chen, Ruwei Ou, Bi Zhao, Wei Song, Xiaojiang Yi, Rong Yang, Xueping Chen

**Affiliations:** ^1^Department of Neurology, West China Hospital, Sichuan University, Chengdu, China; ^2^West China School of Nursing, Sichuan University, Chengdu, China

**Keywords:** Parkinson's disease, the life quality, the acceptance of disability, disability, acceptance

## Abstract

**Background:**

Acceptance of disability (AOD) is a process that a patient must undergo to cope with altered abilities, but its effect in Parkinson's disease (PD) remains unclear. The present study aimed to determine the level of AOD, examine the influence of sociodemographic variables and disease characteristics on the AOD level, and evaluate the relation between AOD level and quality of life in a cohort of PD patients from Southwest of China.

**Methods:**

A total of 336 PD patients were consecutively recruited from November 2018 to October 2020. At enrollment, demographic and clinical data were obtained using a questionnaire, and the Acceptance of Disability Scale-Revised (AODS-R) scale was used to measure the AOD level.

**Results:**

The mean total score of AOD is 87.28, indicating a moderate level of disability acceptance in PD patients. Statistical analysis showed that understanding of PD, family support, and UPDRS-II score were major factors affecting AOD level, and patients with low AOD levels were more likely to have poorer quality of life.

**Conclusion:**

AOD is a serious problem in PD patients in Southwest China, especially among individuals with insufficient family support and a lack of recognition of the disease. AOD was also associated with motor function and daily living ability, thus suggesting that evaluation of the AOD and promotion of the awareness may be helpful to improve the quality of life in patients with PD.

## Background

Parkinson's disease (PD) has become a heavy public health burden in China over the past decades. In 2016, 6.1 million individuals globally had a PD diagnosis, of whom 1.4 million were from China (1). The age-adjusted prevalence rates of PD was more than doubled between 1990 and 2016 in China, which is the largest increase worldwide (1). It is estimated that approximately 8.7 million individuals will be living with a PD diagnosis worldwide, and the number of PD patients in China will rise to 4.94 million in 2030, accounting for 57% of the total number (2).

PD patients typically experience cardinal motor symptoms and numerous non-motor symptoms over a prolonged period. Specifically, neuropsychiatric disorders, such as anxiety and depression, were common in patients with PD, and considered clinically significant in 13–89% of the patient population (3). Our previous study showed that the severity of non-motor symptoms in PD tended to become progressively worse with the course of the disease (4). We also found that non-motor disturbances contributed to the reduced quality of life (QoL) of PD patients with long disease duration (5). Due to the nature of PD, most patients gradually progress to end-stage, and the presence of motor and non-motor complications highly influence patients' QoL (6). The disability increases with disease progression, PD patients would become dependent on care from others. This condition leads to a great negative impact on patients' and caregivers' lives (7), suggesting that the improvement of psychological adjustment would improve the QoL of PD patients.

In general, patients with chronic diseases should receive a psychological intervention program, which is helpful for them to accept the disabilities and adapt to the physical conditions (8). Acceptance of disability (AOD) is considered an important factor that accounts for psychological adjustment improvement (9). To specifically assess AOD, Linkowski et al. developed an AOD scale with 50 items based on the notion of acceptance of loss (10). Moreover, this scale was revised to 32 items by Groomes and Linkowski in 2007. Studies have used this scale to evaluate disability acceptance among patients with chronic diseases, including spinal cord injury (11), cerebral palsy (12), Ehlers-Danlos syndrome (13), diabetes (14), chronic kidney disease (CKD) (9), and stroke (15, 16). The results from these studies indicated that the higher the level of disability acceptance, the better the patients' quality of life.

However, studies on AOD among PD patients are almost blank. To fill this gap, we conducted a study to identify AOD and related factors among PD patients, and examine the relationships between AOD and the QoL of PD patients.

## Methods and Measures

### Assessment of AOD Level

AOD was assessed by the revised version of the AOD scale (AODS-R), which includes 32 items and four dimensions: enlargement of the scope of values (nine items), subordination of physique (five items), containment of disability effect (nine items), and transformation from comparative values to asset values (nine items). Each item is rated on a four-point Likert scale (from “1-strongly disagree” to “4-strongly agree”), and the total score range is from 32 to 128. The higher the score is, the better level of disability acceptance is. The full score is 128; 97–128 is defined as a high acceptance level, 65–96 is moderate, and 32–64 is low. The data collected in the present study yielded a Cronbach's α of 0.83 for the scale (Overall items), and the α coefficients for four dimensions were listed as follows: 0.91 (Transformation), 0.84 (Enlargement), 0.68 (Containment), and 0.66 (Subordination).

### Covariates

The demographic data included sex, age, residence, family history of PD, marital status, educational level, job status, monthly income, and family support. Disease-related characteristics included age of onset, disease duration, the presence of the chronic diseases, the Unified PD Rating Scale (UPDRS) UPDRS part I (17), part II (17), UPDRS part III (17), Hoehn and Yahr (H&Y) stage (18), exercise habits, type of medical insurance, and the knowledge of the illness, which were rated by a questionnaire. UPDRS part I evaluated the patients' mentation, behavior, and mood; UPDRS part II accessed the activities of daily living; UPDRS part III and H&Y stage, which were conducted when patients were in the “ON” medication state, were utilized to evaluate the motor disability. Regular exercise was defined as an exercise for more than 30 min at a time more than five times a week for over three months, including walking, cycling, running, tai chi, dancing, rehabilitation exercises, etc. There are three types of medical insurance: basic medical insurance, commercial insurance, and self-pay. To judge whether patients have an understanding of PD, they were required to answer the following questions: (a) What is PD? (b) What are the treatments for PD? (c) What rehabilitation exercise can you perform for the disease? (d) Do you know the prognosis of the disease? (e) How much do you know about the patient care issues? Patients were asked to choose a number from 0 to 10 to represent their understanding of the disease; the higher score indicated that they knew more disease-related knowledge. The QoL of PD patients was evaluated using PD Questionnaire 8 (PDQ-8), which contained eight items: (1) mobility; (2) activities of daily living; (3) emotional well-being; (4) stigma; (5) social support; (6) cognitions; (7) communication; and (8) bodily discomfort (19–23), ranging from 0 (normal) to 100 (worst disability). The PDQ-8, developed by (24), is a specialized scale for evaluating the quality of life of PD patients (25). The scale has been translated into Chinese and was tested in Singapore and China, and the results showed that the Chinese version of PDQ-8 was verified with good reliability and validity, and the items had high correlation coefficients comparable to those of the English scale (26).

### Study Design and Data Collection

This cross-sectional study was conducted in the Department of Neurology, West China Hospital, Sichuan University. The required sample size was calculated by PASS 11.0 software, and the statistical parameters were set as follows: α = 0.1, effect size (f2) = 0.15, power = 0.9, number of predictors = 30. The calculated sample size was 256 at minimum. At last, a total of 360 PD in-patients were chosen by convenience sampling from November 2018 to October 2020. All PD patients met the United Kingdom PD Society Brain Bank Clinical Diagnostic Criteria (27). Patients with atypical and secondary Parkinsonism, patients who refused to be interviewed, patients who had undergone DBS surgery, and patients who were unable to complete the interview were excluded. This study was approved by the Ethics Committee of West China Hospital of Sichuan University. All participants had provided written informed consent. Before discharge, the trained investigators explained the research purpose, asked questions item by item, and recorded the objective answers. It took 15–20 min to complete the interview.

### Statistical Analysis

All analyses were performed using version 19.0 of Statistical Package for the Social Sciences (SPSS). The continuous data with a normal distribution were listed as the mean ± standard deviation and those with a non-normal distribution were described as the median values (quartile), while the categorical variable was presented as number (percentage). The student's *T*-test was used to compare the continuous data, and the Chi-square test for the categorical variables. Pearson correlation analysis was used to explore the associations between AOD and other continuous variables. Multiple regression analysis was performed to determine the affecting factors of AOD among PD patients. The total AOD score was used as the dependent variable in this analysis. Parameters with significant differences in univariate analysis and factors which have been shown to be significant in previous studies were used as independent variables, including sex, age of onset, family history, family support, work status, monthly income, other chronic diseases, disease duration, regular exercise, insurance type, H&Y stage, UPDRS-I score, UPDRS-II score, UPDRS-III score, and diseases understanding. Multiple regression analysis with QoL as the dependent variable and the identified significant characteristics, including sex, age of onset, place of residence, education level, disease duration, age of onset, regular exercise, monthly income, H&Y stage, diseases understanding, family support, UPDRS-II score, UPDRS-III score, and total AOD score as independent covariables was used to explore the potential factors that may be related to QoL. Collinearity analysis of variance inflation factors (VIF) were carried out to evaluate the possible multicollinearity effects of the influencing factors.

## Results

### Demographic Data and Disease Characteristics

Of the 360 consecutive qualified PD patients, four refused to participate in the survey and 20 were excluded due to an inefficient response rate below 100%. Therefore, data on 336 PD patients were included in the final analysis. The mean age of the included PD patients was 62.55 years (*SD* = 10.55), and 49.4% were men (Table 1). Most patients were from urban areas (72.32%), with low educational levels (below 9th grade) (81.83%). Regarding income, 104 PD patients (31%) were below 3,000 yuan per month (about $470), while 2,210 yuan was the median disposable income for Chinese residents per month in 2019. As for the chronic diseases, 214 PD patients (63.69%) had no other kinds of chronic diseases (Table 1). The disease characteristics were shown in Table 1. The mean UPDRS I score was 1.50 ± 1.97, the mean UPDRS II score was 10.81±6.51, the mean UPDRS III score was 30.64 ± 14.28, and 276 (82.14%) patients were at H&Y stage 1–2.5. Alarmingly, our PD patients showed a lack of understanding of the disease, and the mean score of the disease understanding was 3.83 (Table 1).

**Table 1:** Disease acceptance among PD patients with different demographic and clinic characteristics.

	**Group**	**Number**	**Disease acceptance**	***P-value***
Gender	Male	166 (49.40%)	87.78 ± 8.49	0.275
	Female	170 (50.60%)	86.79 ± 8.17	
Age	30–40	7 (2.08%)	90.43 ± 6.63	0.588
	40–50	37 (11.01%)	88.62 ± 9.55	
	50–60	72 (21.43%)	87.00 ± 9.51	
	60–70	123 (36.61%)	87.44 ± 7.97	
	>70	97 (28.87%)	86.55 ± 7.46	
Residence	Rural	93 (27.68%)	87.40 ± 8.18	0.873
	Urban	243 (72.32%)	87.23 ± 8.41	
Marital status	Married	294 (87.50%)	87.16 ± 8.31	0.752
	Unmarried	7 (2.10%)	87.86 ± 3.13	
	Divorced	8 (2.40%)	90.38 ± 11.96	
	Widowed	27 (8.00%)	87.48 ± 8.50	
Family history	No	302 (89.88%)	87.17 ± 8.34	0.455
	Yes	34 (10.12%)	88.29 ± 8.30	
Family support	No	98 (29.17%)	85.64 ± 6.72	**0.021**
	Yes	238 (70.83%)	87.95 ± 8.84	
Work status	No	289 (86.01%)	87.46 ± 8.04	0.316
	Yes	47 (13.99%)	86.15 ± 9.99	
Education level	Primary school or blow	115 (34.23%)	87.09 ± 8.44	0.849
	junior-senior high school	160 (47.60%)	87.21 ± 8.08	
	Bachelor degree or above	61 (18.20%)	87.82 ± 8.33	
Monthly income	<3,000	104 (31.00%)	88.72 ± 7.79	**0.034**
	≥3,000	232 (69.00%)	86.63 ± 8.49	
Chronic diseases	None	214 (63.69%)	88.00 ± 8.25	0.424
	≥1	122 (28.87%)	87.76 ± 8.47	
Age of onset	≤50	89 (26.49%)	88.74 ± 9.09	0.053
	>50	247 (73.51%)	86.75 ± 8.00	
Disease duration	≤36	154 (45.83%)	86.53 ± 8.39	0.127
	>36	182 (54.20%)	87.92 ± 8.24	
Exercise regularly	No	127 (37.80%)	87.61 ± 8.18	0.576
	Yes	209 (62.20%)	87.08 ± 8.44	
Insurance type	Basic medical insurance	325 (96.73%)	87.11 ± 8.30	0.117
	Commercial insurance	6 (1.79%)	91.83 ± 10.27	
	Self-pay	5 (1.49%)	93.00 ± 5.61	
Hoehn-Yahr	1–2.5	276 (82.14%)	86.67 ± 8.22	**0.004**
	3–5	60 (17.90%)	90.08 ± 8.30	

*The bold values: P < 0.05*.

### AOD Level and the Differences Among PD Patients With Different Demographic and Clinic Characteristics

The mean AOD score was 87.28 ± 8.33, and 55.3% of the PD patients scored at a low AOD level (below the median of 86.00). The ranking of standardized scores of four dimensions was shown in Table 2, the scores of “Enlargement” and “Transformation” were the highest, and “Subordination” was the lowest scoring dimension. When comparing the AOD scores in PD patients with different demographic data and disease-related characteristics, the differences were of no statistical significance among PD patients with different gender, age of onset, disease duration, place of residence, marital status, family history, chronic diseases, exercise habits, job status, educational levels, and type of medical insurance (*P* > 0.05). Patients with higher AOD levels were more likely to have lower monthly income, better family support, more advanced H&Y stage, and a better understanding of the disease (Table 1). The results of Pearson correlation analysis showed that the AOD level was associated with UPDRS-II score (*P* < 0.001) and UPDRS III score (*P* < 0.001), but not UPDRS-I score (*P* = 0.07).

**Table 2:** Total AOD score and each dimension score among PD patients.

	**Mean**	**Standard deviation(SD)**	**Standardized scores^#^**	**Minimum-maximum**
Total AOD score	87.28	8.33	100.00	59-123
Enlargement	24.12	3.88	28.12	12-36
Subordination	14.84	1.88	15.62	9-20
Containment	23.69	4.70	28.12	9-36
Transformation	24.58	2.98	28.12	15-36

# *Standardized scores = (mean ÷ total score) × 100%*.

### The Effects of Other Variables on AOD and Its Correlation to QoL

Multiple linear regression was employed to analyze influencing factors of AOD level in our PD patients, and the results in Table 3 demonstrated that three variables, including the understanding of PD, care situation, and UPDRS-II score, significantly affected PD patients' AOD level. VIF values were varied between 1.009 and 1.059, and the multi-collinearity problems were absent. Besides the place of residence, disease duration, H&Y stage, diseases understanding, UPDRS-II score, and UPDRS-III score, the total AOD score was correlated positively with the PDQ-8 scores and disease acceptance significantly affected the QoL of PD patients (Table 4).

**Table 3:** Multiple linear regression analysis for the AOD level.

**Independent variable**	***β value***	***T value***	***P value***	***VIF***
UPDRS-II	0.317	6.066	**0.000**	**1.059**
Disease understanding	0.183	3.582	**0.000**	**1.009**
Family support	0.153	2.994	**0.003**	**1.009**

*The bold values: P < 0.05*.

**Table 4:** Multiple linear regression analysis of PDQ-8.

**Independent variable**	**The standard coefficient β**	***T value***	***P value***	***VIF***
Total AOD score	0.277	5.814	**<0.001**	**1.117**
UPDRS-I	0.197	4.398	**0.000**	**1.102**
UPDRS-II	0.268	3.577	**0.000**	**1.671**
Disease duration	0.111	2.382	**0.018**	**1.185**
Residence	−0.124	−2.743	**0.006**	**1.117**
Hoehn-Yahr	0.101	1.977	**0.049**	**1.429**
Diseases understanding	−0.116	−2.533	**0.012**	**1.157**

*The bold values: P < 0.05*.

## Discussion

In the present study, we were trying to determine the level of AOD and its effects on QoL in PD patients and determine the influence of sociodemographic variables and disease characteristics on AOD. To our knowledge, this is the first study to examine AOD and its correlates among patients with PD in China. We found that the mean total ADD score of our PD cohort was 87.28 (*SD* = 8.33), and 55.3% of patients scored below the median of 86.00. This score was higher than that in stroke patients (Mean = 71.72 and 74.15, respectively) (15, 16), but similar to that in patients with CKD (Mean = 85.02). A previous study from Poland used the acceptance of Illness Scale (AIS) to evaluate the level of acceptance of the disease in 50 PD patients, and found that 62% of participants had a moderate acceptance level (28). PD is a progressive neurodegenerative disease, with a long disease duration. A meta-analysis found that patients typically lived 6.9 to 14.3 years after a diagnosis of PD, and the Sydney multicenter study found that 36 of 136 patients (26%) diagnosed with PD lived for at least 20 years (29, 30). Similarly, studies found that patients with chronic neurological diseases or chronic diseases, including PD, CKD, and osteoarthritis, had a moderate level of acceptance. The present study's finding indicates that the AOD of PD patients is at a medium level but still needs to be improved. However, the relatively poor AOD among half of the PD patients was not unexpected. First, access to rehabilitative resources is limited in developing countries, while these resources are common in developed countries, including outpatient rehabilitation, support groups, pain management, and psycho-educational programs. It is reasonable that PD patients who have low acceptance of their disabilities should receive standard rehabilitation training to maximize functional abilities. Second, the conduction of AOD evaluation is lacking, and the caregivers do not realize that encouragement on positive self-evaluation is essential. Therefore, standardized AOD assessment in routine care may help PD patients receive further therapeutic interventions.

We also tried to determine the influence of sociodemographic variables and disease characteristics on AOD in PD patients, and found that factors, including family support, UPDRS-II scores, and the understanding of the disease, had effects on the AOD level. The care from the family had a positive effect on the AOD of PD patients, and this result was in line with the findings in stroke patients (16). Currently, in China, caring for PD patients mainly depends on family support. A PD patient always manages personal affairs with the aid of a support network formed by family members. It is thought that people who enjoy a higher degree of support have better outcomes, including physical and mental health (31). Similarly, a previous study also found that PD patients from regional, rural and remote areas received the most support from family, and few received any community supports (32). The care situations of our PD patients may be related to the economic level, and a more advanced and sound care system from family, hospital, and community should be established. In higher-income country contexts, the importance of maintaining social relations and maintaining association through support groups in PD patients has been widely reported (33). The full supports can provide a way to gain information, enable continued engagement inactivity, and provide social support connection (34). Social support could significantly influence self-efficacy in managing chronic illness (35), and PD patients also need continuous empowerment through supportive personal relationships to better QoL. Patients with PD who had higher UPDRS-II scores scored higher on AOD, implying that impaired daily activities may play a predominant role affecting AOD level among PD patients. It is speculated that PD patients who experience more apparent symptomatology would become calmer to deal honestly with themselves, and act openly to the worst aspects of physical disability, and thus become preoccupied with acceptance. However, a previous study evaluated the functional capability using instrumental activities of daily living (IADL) scale and found that more independent PD patients were more likely to accept their disease (28). Studies in stroke patients also observed the low levels of disease acceptance in patients with a poorer functional condition (36). The inconsistent findings may be partially due to differences in sample size, age distribution, assessment scale, and region and race. A well-designed study with larger samples is required to verify the impact of daily living ability on AOD. In the present study, a higher level of disease awareness correlated with a higher AOD level in PD patients. Patients with an improved understanding of their disease would be more capable of mediating emotional and behavioral responses provoked by negative events. Therefore, patient education is important, and patients need to be equipped with knowledge of the diseases. We also found that patients in a more advanced stage of disease had higher AOD levels, which was consistent with a previous argument that AOD studies were more meaningful when conducted in the context of patients with chronic diseases (37). We speculate that PD patients in the more advanced stage have gradually learned to accept the consequences and adjust to life changes during the chronic course. Therefore they have higher AOD scores. We also found that PD patients with the H&Y stage of 1–2.5 had lower AOD scores than those with higher stages. We recommend that greater attention be paid to newly-diagnosed PD patients to facilitate early referral for further therapeutic interventions. The current study results showed that the AOD level in our PD patients affected the QoL of patients; the AOD level was positively correlated with QoL. Similarly, a study from Poland also found that disease acceptance significantly affected QoL (28). Higher AOD level was also found to contribute significantly to higher overall QoL in patients with colostomy (38), facial burn scar (39), epilepsy (40), and stroke (41). First, emotionally, improvement in AOD level changes the cognition of PD patients, promotes their control over their own emotions, allows them to perceive more positive psychological emotions, and thus their attitude toward life. Second, physically, a good attitude and logical thinking are helpful to overcome the belittling of self-esteem and obey their current physical conditions. If the PD patients improved their AOD, they would be more aware of their abilities and be more adaptive to promote the physical condition.

The initial investigation provides firsthand evidence of the applicability of the AODS-R scale among PD patients in China. However, several limitations should be considered when interpreting the results of the current study. First, the PD patients were recruited from a single hospital in southwest China, so the findings did not apply to all PD patients. Second, the current study did not measure some factors, including religious beliefs, family dynamics, emotional support, and environmental factors. It was found that stroke patients who reported having religious beliefs had higher AOD scores than those who did not turn to religion (15). Furthermore, disability acceptance and emotional support from friends partially mediated the relationship between functional disability and QoL in individuals with serious mental illness (42). Third, the study was cross-sectional, and AOD scores may change over time. Longitudinal studies are required to examine the dynamic change of AOD level over time and explore further causal relationships among the affecting factors.

## Conclusions

The AOD level among PD patients in China was moderate, and it was influenced by family support, disease severity, and understanding of the disease. All-round support is vital for adaption, and education programs should be developed on the theoretical foundation. In addition, AOD level is an independent factor affecting the quality of life in PD patients, indicating that the quality of life of PD patients will improve with the improvement of disability acceptance level.

## Data Availability Statement

The original contributions presented in the study are included in the article/supplementary material, further inquiries can be directed to the corresponding author.

## Ethics Statement

This study was approved by the Ethics Committee of West China Hospital of Sichuan University. All participants had provided written informed consent.

## Author Contributions

XC and RY conceived this study. DC, RO, BZ, WS, and XY did the patient evaluation and data collection. YL did the statistical analysis. XC and YL wrote the manuscript while all the authors revised and discussed the final edition.

## Funding

This study was supported by grant from National Key Research and Development Program of China (Grant No. 2017YFC09007703), grant from Science and Technology Planning Project in Sichuan Province (Grant No. 2020YJ0281), grant from West China Nursing Discipline Development Special Fund Project, Sichuan University (Grant No. HXHL21042), and the grant from Health Department of Sichuan Province (Grant No. 18PJ268).

## Conflict of Interest

The authors declare that the research was conducted in the absence of any commercial or financial relationships that could be construed as a potential conflict of interest.

## Publisher's Note

All claims expressed in this article are solely those of the authors and do not necessarily represent those of their affiliated organizations, or those of the publisher, the editors and the reviewers. Any product that may be evaluated in this article, or claim that may be made by its manufacturer, is not guaranteed or endorsed by the publisher.
